# Supplementary material for: Phage-inducible chromosomal minimalist islands (PICMIs), a novel family of small marine satellites of virulent phages
Source: Nat Commun. 2024 Jan 22;15:664. doi: 10.1038/s41467-024-44965-1 (PMC10803314; doi:10.1038/s41467-024-44965-1)
Supplement: Supplementary file 1 — Supplementary information [file 41467_2024_44965_MOESM1_ESM.pdf]

Phage-inducible chromosomal minimalist islands (PICMIs), a novel family of small marine satellites of virulent phages

SUPPLEMENTARY INFORMATION

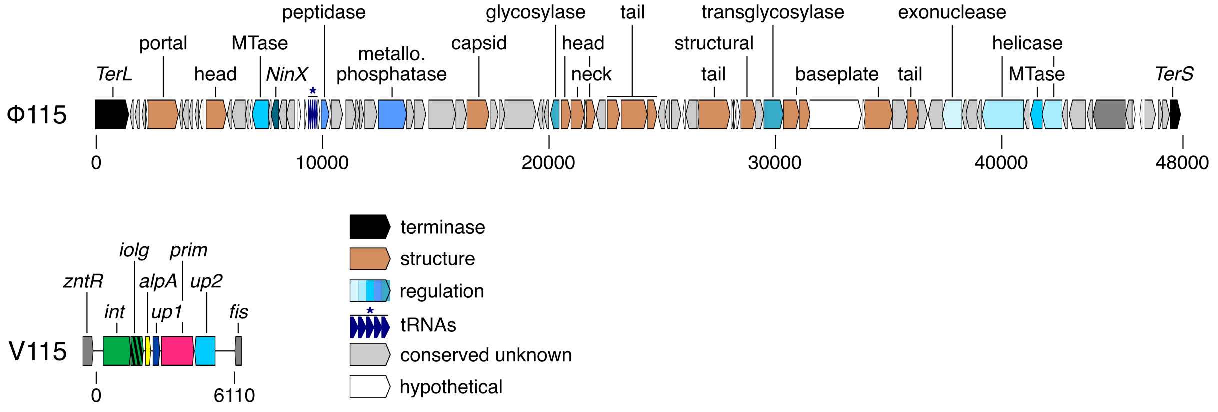

Supplementary Figure 1. Genome assembly and annotation of the phage 115_E_34-1 (Φ115) revealed two contigs of 47,851 and 6,110 bp respectively. The larger contig corresponds to the complete genome of the phage, ordered here from the large (*TerL*) to the small (*TerS*) terminase (locus tags VP115E341_P0001 to 0093). The small contig were also found in the genome of the host used to isolate and propagated this phage, *V. chagasii* 34_P_115 (V115). This region is integrated in the bacterial genome at the end of the *fis* regulator gene (locus tag VCHA34P115_150113) and flanked by two direct repeats of 17 bp (aatacggcatgaactaa). Among the six genes identified in this region, *int* encodes for an integrase, *alpA* for a putative regulator and *prim*, a putative primase.

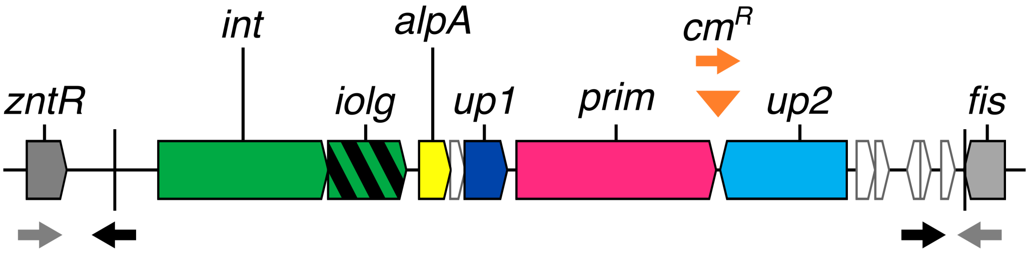

Supplementary Figure 2. Syntactic annotation of the satellite using Phanotate without size threshold suggest six additional putative genes (in white) that were considered as false open reading frame (ORF).

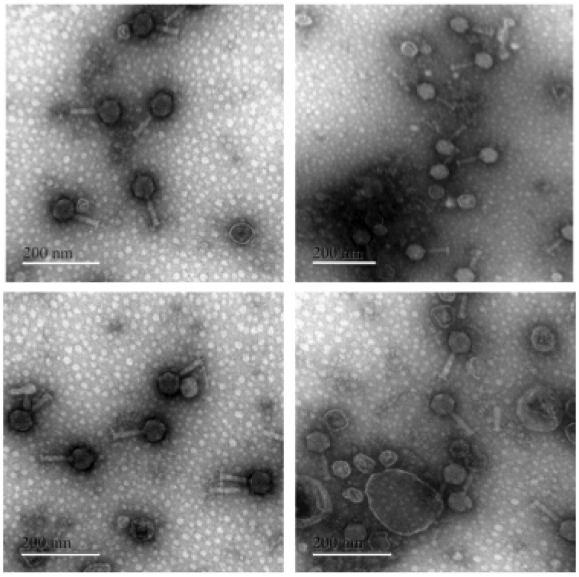

Supplementary Figure 3. Transmission electron micrographs of Φ115 myovirus, produced using *V. chagasii* V115 as host. Images are representative of the observation of dozens of particles that did not revealed heterogeneity in capsid size. Images were collected using a Jeol JEM-1400 TEM Microscope. Scale bars are 200 nm.

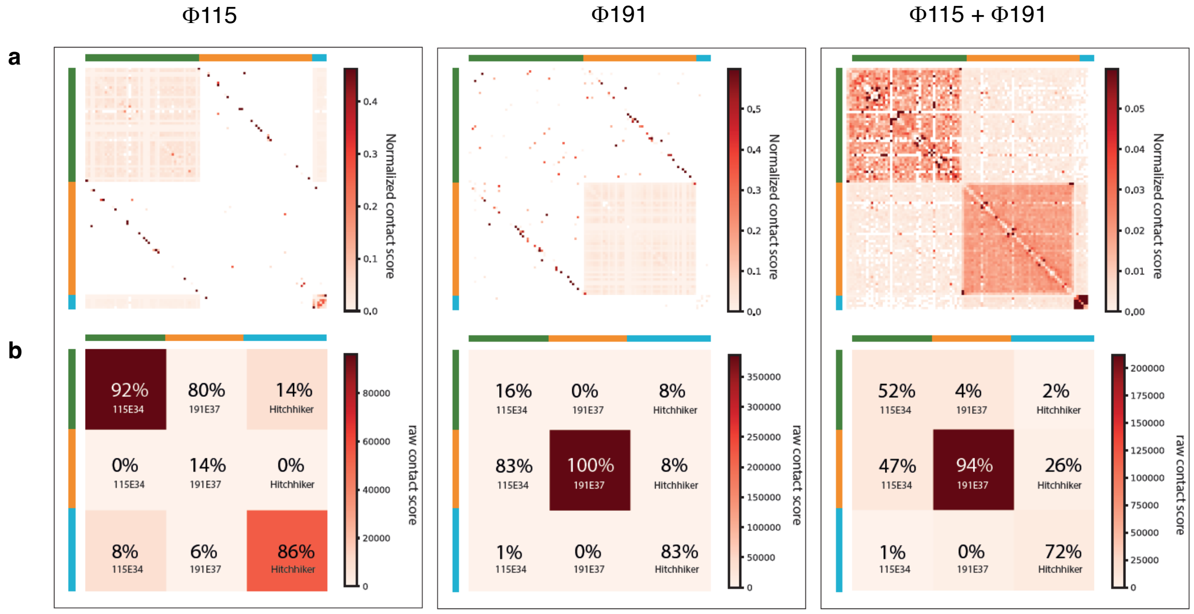

Supplementary Figure 4. HiC experiment showed that the genome of the phage Φ115 and its satellite are located in distinct viral particles. **a** Chromosomal contact maps of phage Φ115, phage Φ191 another myovirus with similar genome size and a single genome, or a mixture 1:1 of both phages. The color code representing normalized contacts between DNA regions from low (white) to high (red) frequencies (1pixel = 1kb) (color scale: vmax = 99% of maximal value). The genome sequence of the helper phage Φ115 and its satellite is indicated in green and blue respectively. Genome sequence of the phage Φ191 is in orange. The result supports the absence of physical contact between the phage Φ115 and the satellite DNAs. The observed contacts result from background. All three genomes show a signal in the upper right and lower left corners indicating circular molecules or a circular permutation phenomenon. **b** Contact map representing the raw contact signal for the same three experiments. Proportions of contact in each category are indicated in percentage relatively to total number of contacts for each genomic entity.

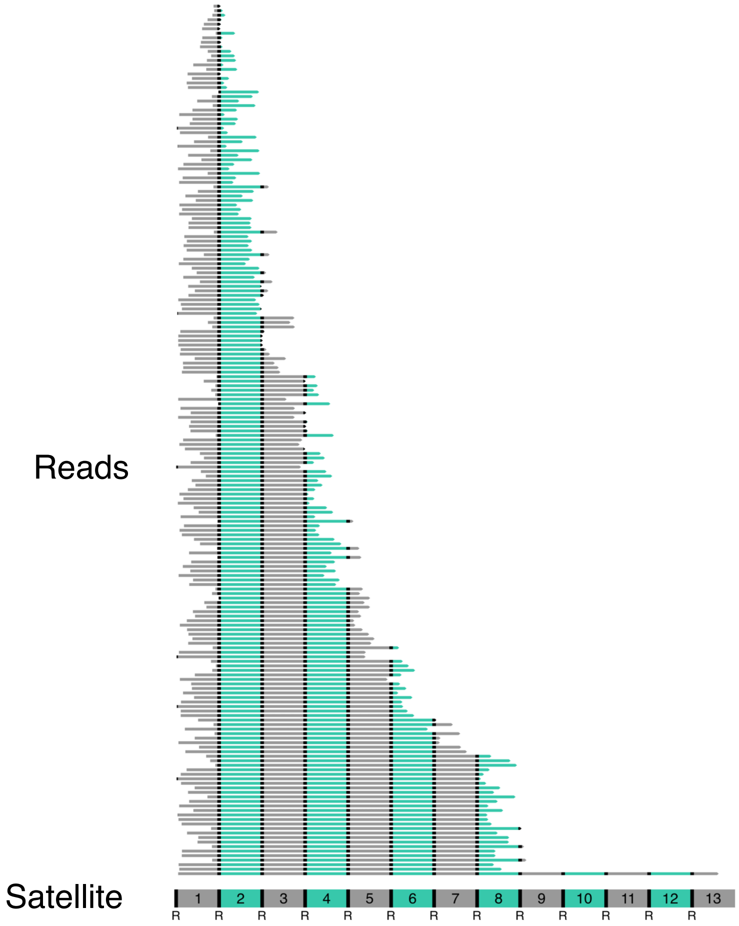

Supplementary Figure 5. Nanopore reads mapped to PICMI$_{115}$ concatemer structure. Nanopore sequencing of Φ115 revealed that a fraction of the viral particles contained a concatemer of 8 copies of the 6.1 kbp satellite leading to ~49 kbp size. All obtained reads were aligned with FAMSA to an artificial PICMI$_{115}$ concatemer of 13 copies (corresponding to the largest read length). Each copy of PICMI$_{115}$ concatemer are represented with an alternance of grey/green and overlapping sequences correspond to the direct repeats (R).

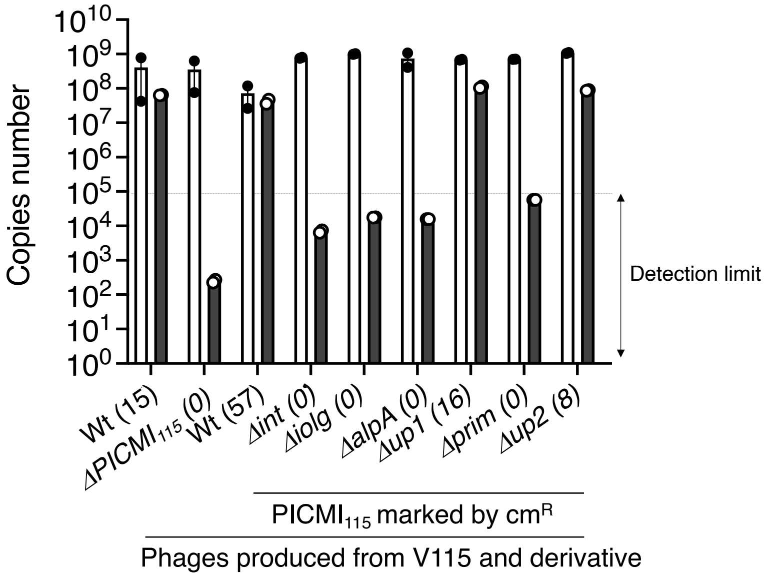

Supplementary Figure 6. Estimating the proportion of viral particles within a population that contain phage satellites rather than phage DNA**.** The number of copies of phage Φ115 (white bar) or octamer of the satellite (black bar) were determined by qPCR using DNAs extracted from a high titer of phages produced in V115 wild type (wt) and a derivative lacking the entire satellite ($\Delta PICMI_{115}$). Source data are provided as a Source Data file. For transduction experiments, a chloramphenicol resistance marker (Cm^R) was introduced within the satellite of V115 and derivatives lacking one of the six satellite genes (e.g., Δ*int*). Bar charts show the mean +/- Standard error of the mean (SEM) from two technical replicates experiments (individual dots). On the x axis, number in brackets indicates the estimate percentage of the Φ115-like particles hitchhiked by the satellite. The dashed line indicates the limit of detection for this assay.

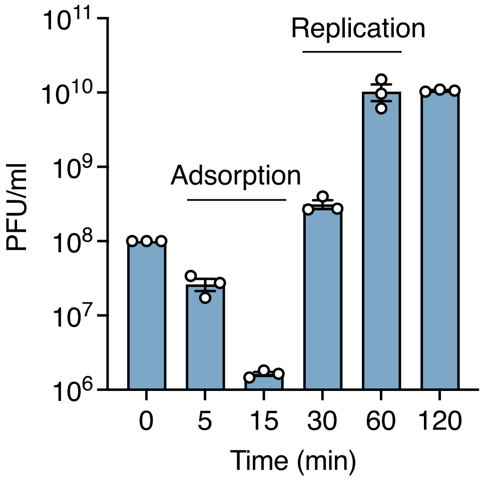

Supplementary Figure 7. Estimation of the infection dynamic by Φ115. *V. chagasii* strain V115 was grown to mid-exponential phase in Marine broth (OD=0.3) and infected with pure Φ115 at a multiplicity of infection (MOI) of 10. At the indicated times, aliquot of the culture was centrifuged, the supernatant was filtered at 0.2 µm and the titer of phages was determined by drop spotting serial dilutions of the supernatant on the host lawn. Bar charts show the mean +/- SD from three independent experiments (individual dots).

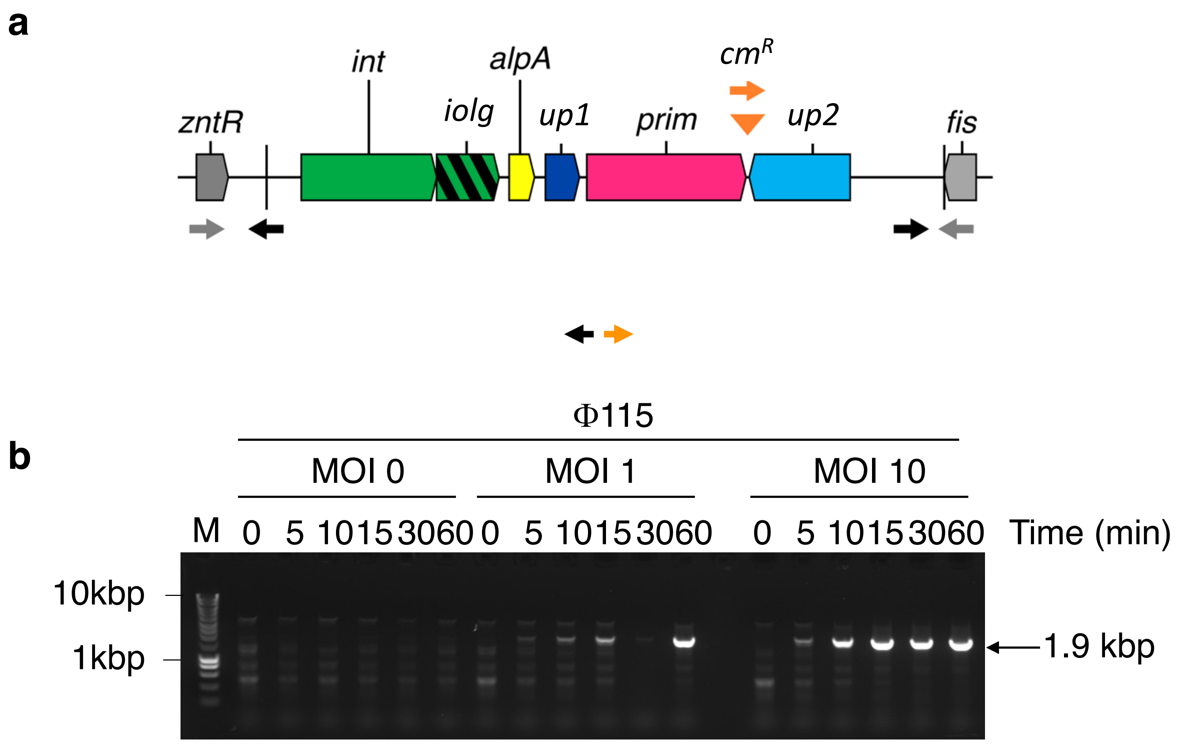

Supplementary Figure 8. PICMI$_{115}$ is induced following Φ115 infection. **a** Schematic representation of PICMI$_{115}$ integrated between the *fis* and *zntR* genes in the genome of *V. chagasii* strain V115. For transduction assays, this element was marked by a chloramphenicol resistance cassette (orange triangle). Arrows depict Forward and Reverse primers used to detect the circularized and concatemeric form of PICMI$_{115}$ (1972 bp). **b** A phage Φ115 stock was produced using V115 as host and thus contains ~15% of particles containing PICMI$_{115}$. The V115 host strains was grown to mid-exponential phase in Marine broth (OD=0.3) and infected with Φ115 at a multiplicity of infection (MOI) of 0, 1 and 10. At the indicated times (in minutes) cells were pelleted, total DNA was extracted and used as template for PCR to detect the circularized and concatemeric form of PICMI$_{115}$. Uncropped gel is provided as a Source Data file.

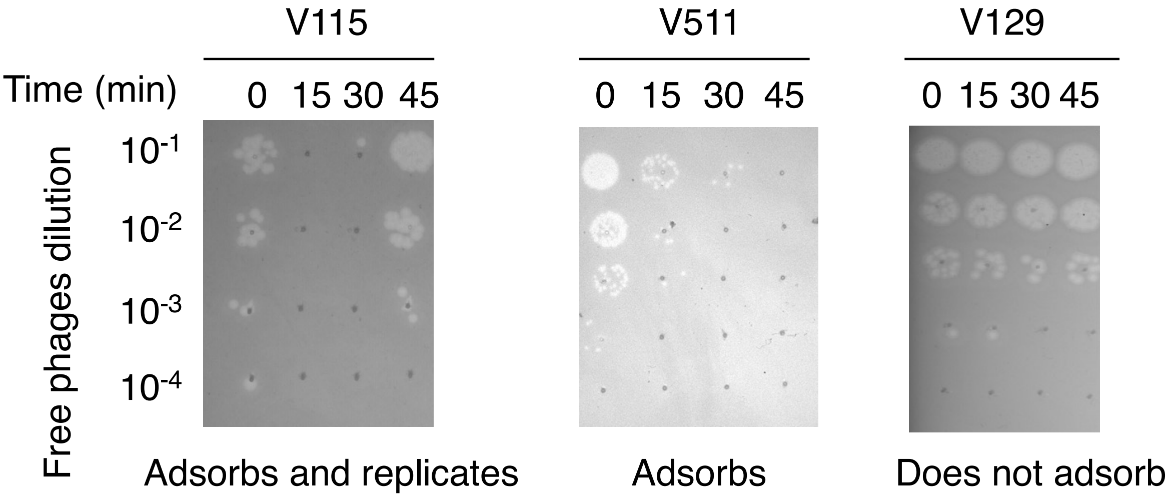

Supplementary Figure 9. Estimation of the adsorption of Φ115 on three *V. chagasii* strains. After allowing a fixed concentration of phages (MOI 0.01) to adsorb to each *V. chagasii* strain for the indicated time, free phages that remained unattached were serially diluted and plated with the original host, V115. In this assay, a drop in the number of infectious particles indicates phage adsorption. Adsorption was complete after 15 or 30 minutes using respectively V115 and V511 as host and was not observed up to 45 minutes using V129. After 45 minutes, production of phages was observed only when using V115.

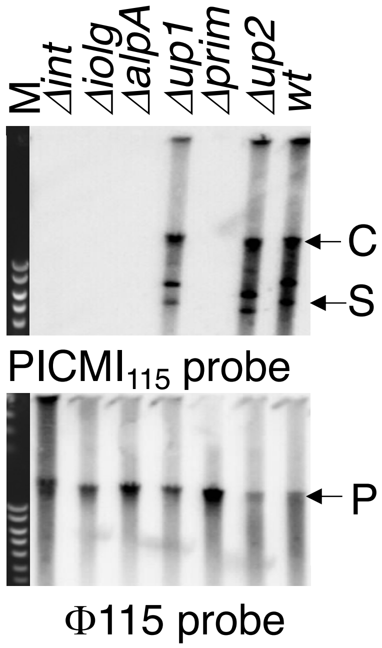

Supplementary Figure 10. Genes necessary for $PICMI_{115}$ activation. The strain V115 and derivatives lacking one of the six genes from $PICMI_{115}$ were infected by the phage Φ115pure for 30 minutes. DNA was extracted from bacteria separated on a 0.7% agarose gel and Southern blotted with $PICMI_{115}$ or Φ115 probes. M: molecular marker (Smart ladder Eugentec). C, S and P indicate the concatemeric and single form of $PICMI_{115}$ and phage genome respectively. Uncropped gel and blots are provided as a Source Data file.

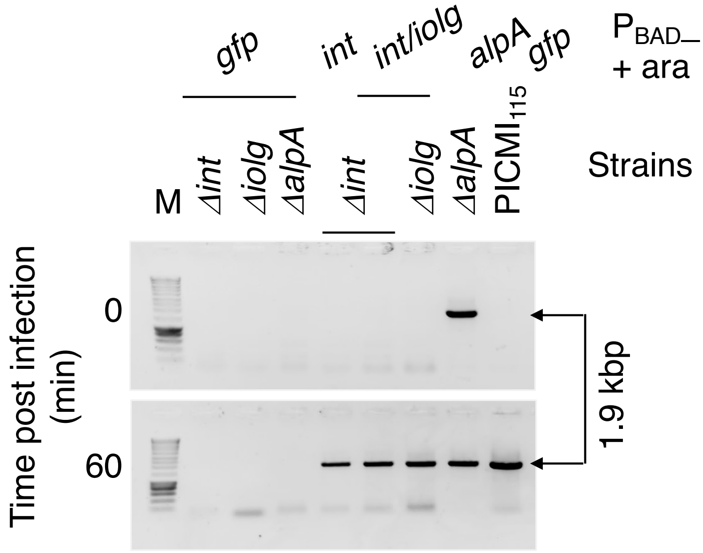

Supplementary Figure 11. For complementation assays, the genes necessary for $PICMI_{115}$ excision, *int/iolg* and *alpA* or, as control, *gfp* were cloned under the control of the arabinose inducible promoter P_{BAD} and transferred to their respective mutants. Strains were grown to mid-exponential phase in the presence of 0.2% arabinose and then infected with Φ115pure for 60 min. Circular and concatemeric form of $PICMI_{115}$ was detected by PCR and separation on SYBR green stained agarose gel. Uncropped gel is provided as a Source Data file. The expression of *alpA* is sufficient to induce $\Delta PICMI_{115}$ activation in the absence of phage (time 0 minutes).

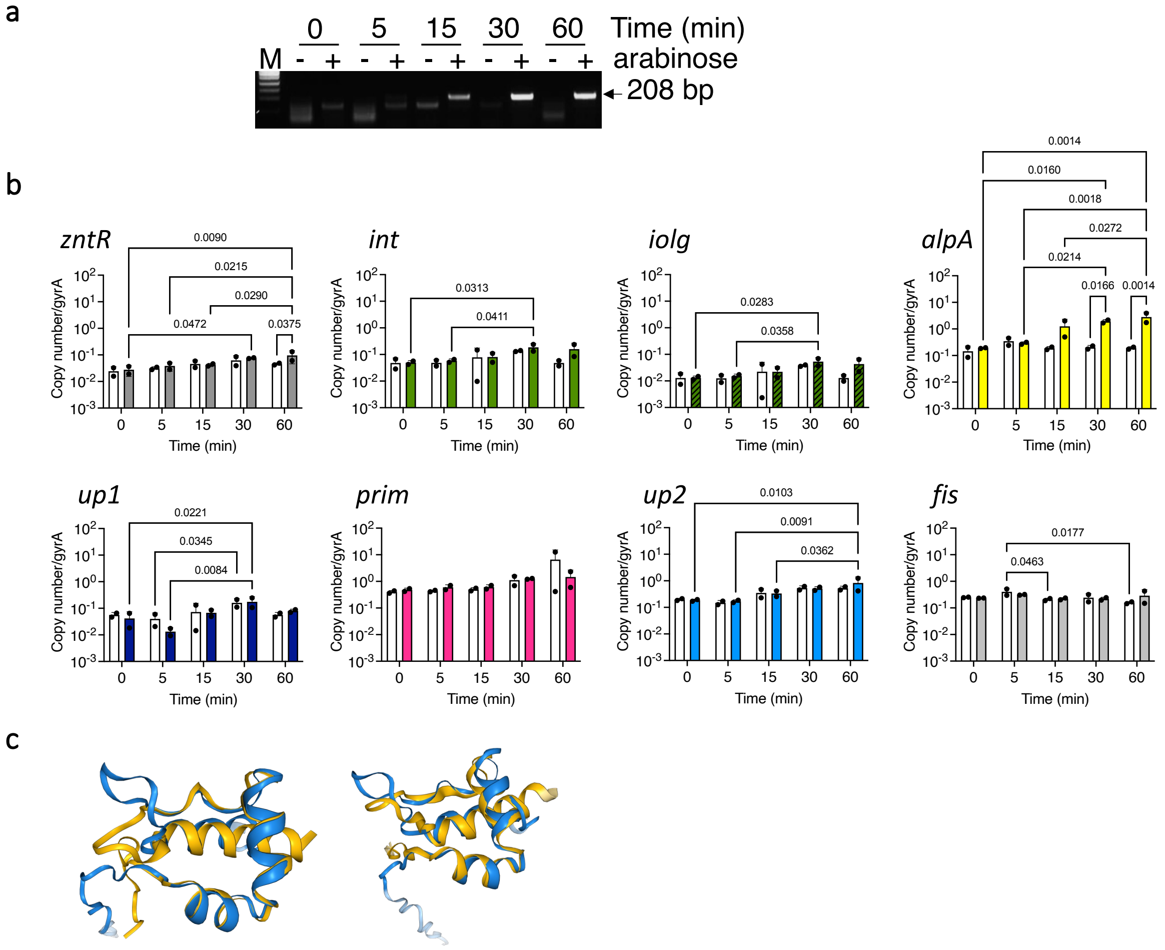

Supplementary Figure 12. AlpA is rather involved in the formation of the excision complex than a transcriptional regulator of PICMI115 genes. The gene *alpA* cloned in a plasmid under the control of the conditional P_{BAD} promoter was transferred into the *ΔalpA* mutant. The strain was grown to mid-exponential phase in Marine broth (OD=0.3) and arabinose (0.2%) was added (+) or not (-) as indicated. At the indicated time, total DNA and RNA were extracted. **a** PCR and gel stained to detect the circularized and concatemeric form of PICMI115. Uncropped gel is provided as a Source Data file. **b** qRT-PCR to detect the expression of each of the six genes from PICMI115, as well as the two flanking genes *fis*, *zntR* and the house keeping gene *gyrA*. The resulting copies number were normalized on *gyrA*. Bar charts show the mean +/- SD from two independent experiments (individual dots). A two-way ANOVA with uncorrected Fisher's LSD was conducted to assess mean differences across all genes and time points. Only pairwise comparisons with a P value < 0.05 are presented. The analysis revealed that, among PICMI genes, only *alpA* expression was influenced by arabinose, as anticipated due to its expression in *trans* from the pBAD promoter. **c** Structure superposition of AlpA (blue) and TorI response regulator (left, yellow) and Xis excisionase (right, yellow). AlpA from PICMI structure is predicted using ColabFold (v1.5.2) and search against AlphaFold/Swiss-Prot v4 database using Foldseek (v7-04e0ec8). The top 2 hits are an excisionase (P15482) from *Streptomyces ambofaciens* (TM-score=0.653) and a TorI response regulator inhibitor for tor operon (Q2EES9) from *Escherichia coli* strain K12 (TM-score=0.625).

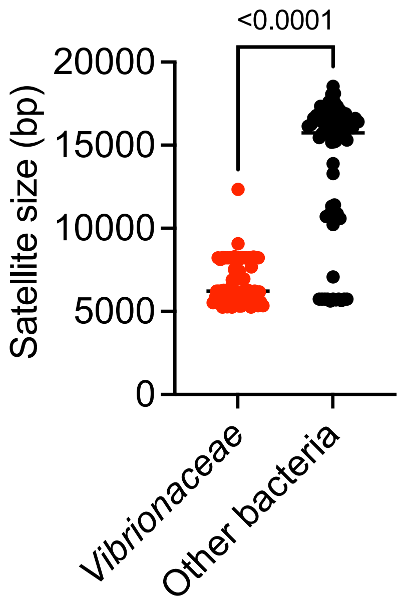

Supplementary Figure 13. Comparison of the size of the 135 putative satellites, identified by the colocalization of *int, alpA, prim* and *fis* genes, in all Genbank bacterial genome (v243), including 67 elements found in *Vibrionaceae* genomes. P value from unpaired t test is indicated.

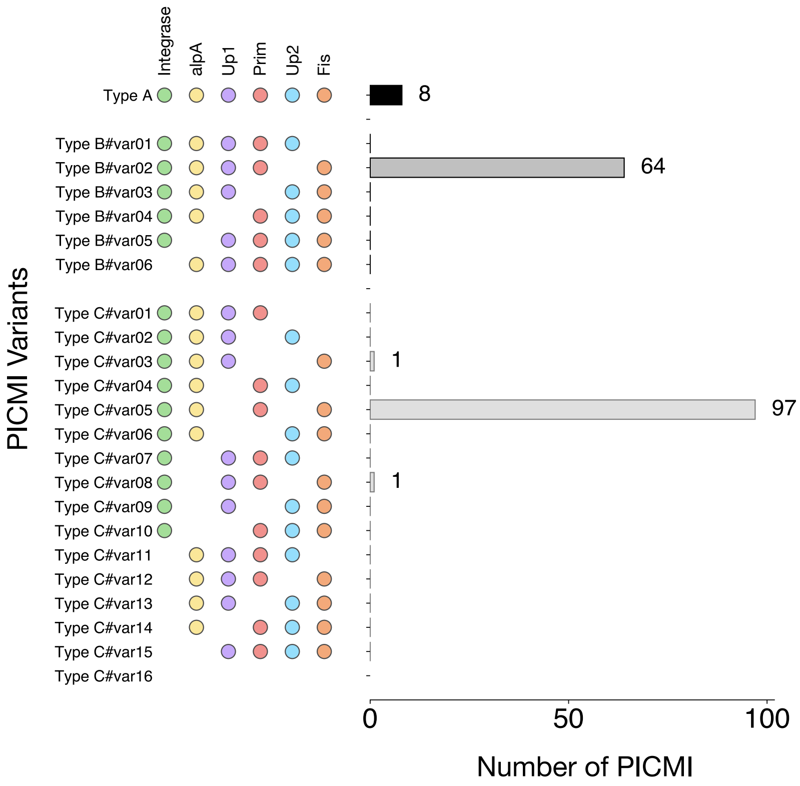

Supplementary Figure 14. Number of the different variants of PICMI elements identified in bacterial genomes. Each circle represents a core component of the PICMI used in the SatelliteFinder model. The absence of a circle (in rows) corresponds to a PICMI variant where a particular component (for Type B) or two components (for Type C) is, or are, undetected.

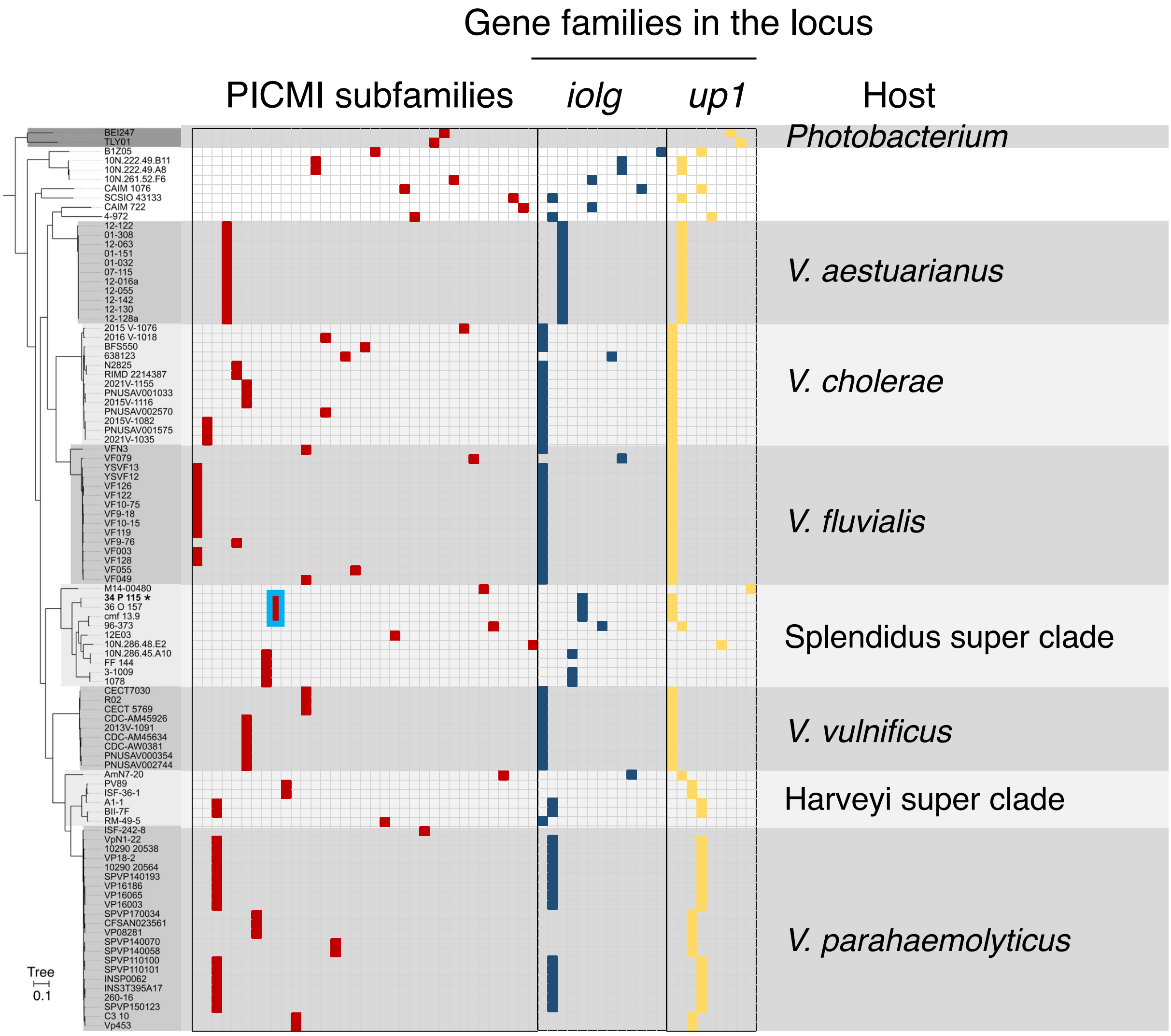

Supplementary Figure 15. Genes that are frequent across the PICMI subfamilies found in the *Vibrionaceae*. Persistent core phylogenetic tree was constructed using PanACoTA (90% for the persistent genome resulting to 1026 families and 30% for minimum percentage of identity) with iqtree2 (1000 bootstrap and GTR model). Among the 35 PICMI subfamilies (35 columns, brown squares), 21 carry an integrase overlapping gene (*iolg*) and four elements carry a gene contiguous to the *int* gene (*icg*). The *iolg* or *icg* were grouped in 13 distinct gene families (13 columns, dark blue squares). Together with the core genes *alpA* and *prim, up1* is part of the early regulon activated by the helper phage. Homologs of PICMI$_{115}$ *up1* were found in 12 PICMIs. Some single genes from eight distinct families, encoding for unknown function, were also present between *alpA* and *prim* in other PICMIs (total 9 gene families in the locus where we found *up1* in PICMI$_{115}$). The subfamily corresponding to PICMI$_{115}$ is framed in blue.

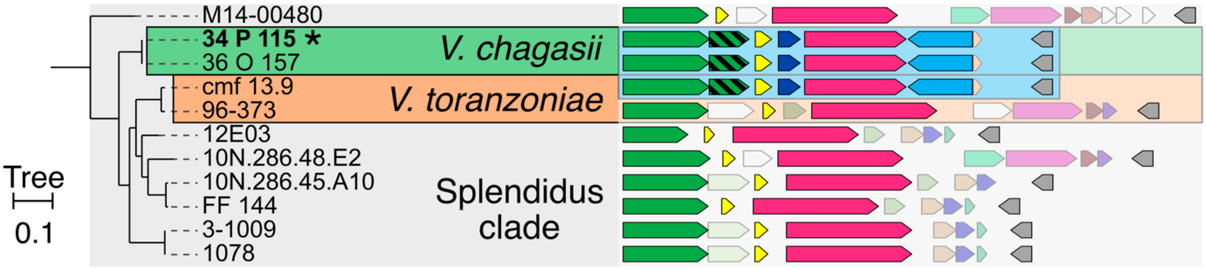

Supplementary Figure 16. The PICMI115 subfamily was detected in two strains of *V. chagasii* (34_P_115 and 36_O_157 named V115 and V157 for simplicity) and in a *V. toranzoniae* strain (cmf 13.9) within the Splendidus clade (zoom in the phylogenetic tree from Fig. 5). Another *V. toranzoniae* (96-373) carries a different PICMI subfamily.

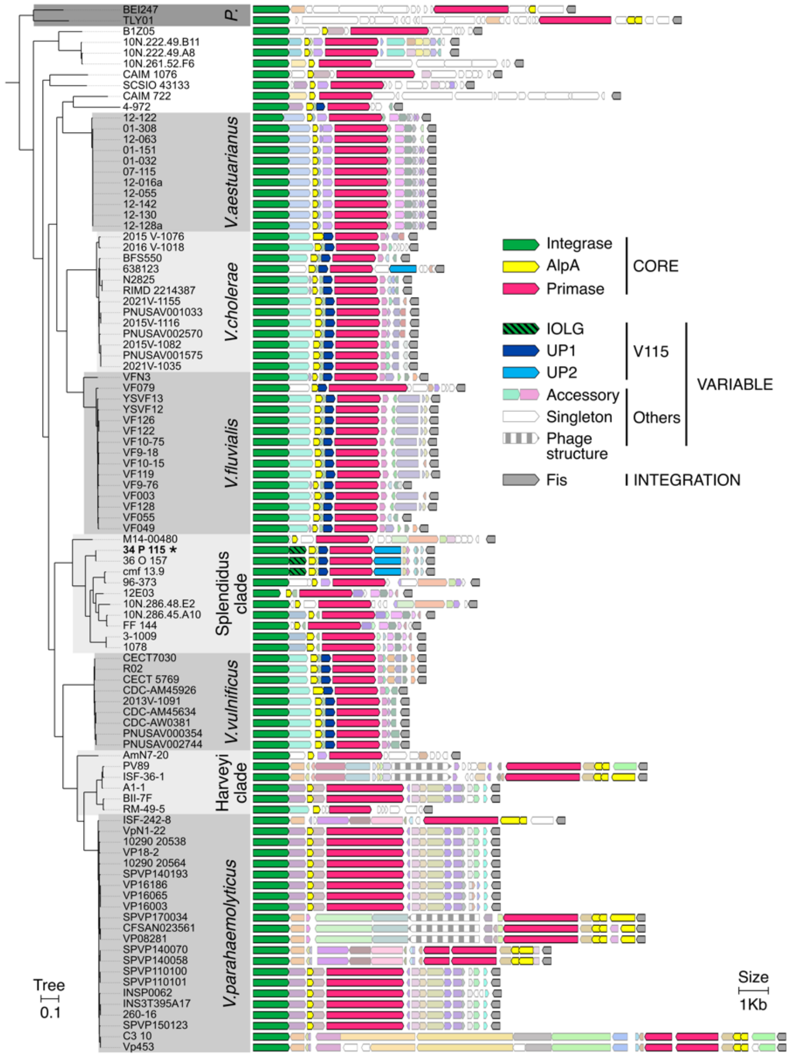

Supplementary Figure 17. Phylogenetic persistent core tree and genomic representation of the 97 PICMI elements found in *Vibrionaceae* (GenBank 01-27-23 containing 19189 organisms). Persistent core tree was constructed using PanACoTA (90% for the persistent genome resulting to 1026 families and 30% for minimum percentage of identity) with iqtree2 (1000 bootstrap and GTR model). Genus, super clades or of species names are indicated in the grey boxes. "P." correspond to *Photobacterium* genus, Harveyi, Splendidus are super clades encompassing several *Vibrio* species. The PICMI$_{115}$ element is pinpointed by bold strain name (34_P_115, V115) and by an asterisk. PICMI-like elements were searched using SatelliteFinder and the identified genomic regions were reannotated using phanotate without length filter. This led to the syntaxic annotation of several small ORFs (<50 amino acid), including in PICMI$_{115}$, that were considered as fragmented and/or pseudogenes. The region from the integrase to the *fis* gene was plotted using the DnaFeaturesViewer python library. Solid colors indicate core PICMI$_{115}$ genes. Grey colors indicate accessory and singleton PICMI-like genes defined using reciprocal best-hit with 20% identity for 50% coverage.

a

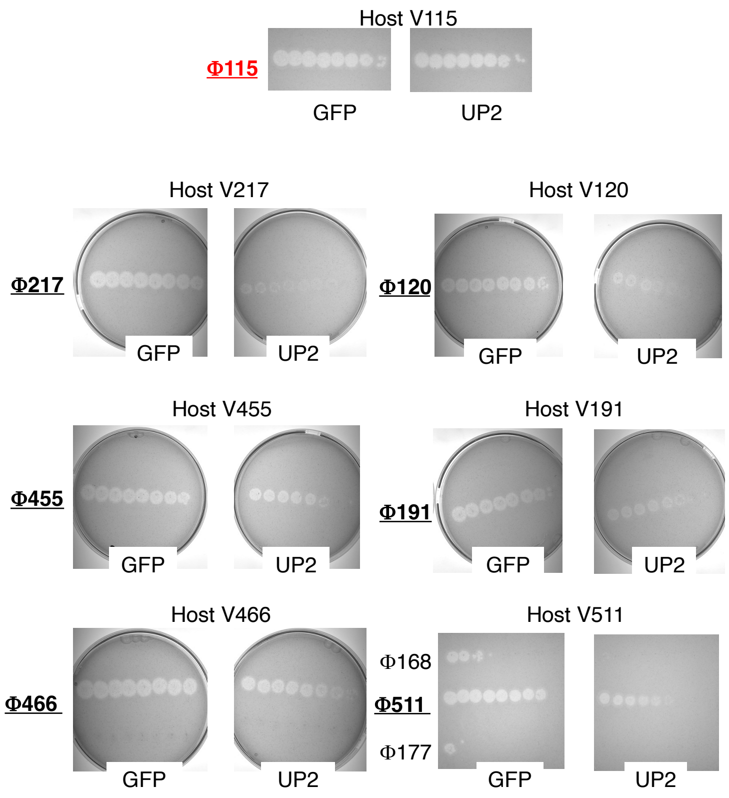

b

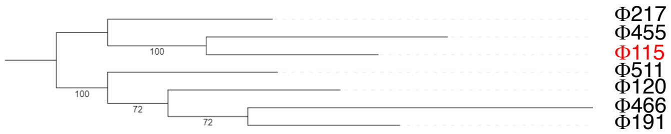

Supplementary Figure 18. Changes in susceptibility to phage killing observed for *up2* expressing *V. chagasii*. **a** Tenfold dilutions of the indicated phages (bold underlined, from the same VIRIDIC family) were spotted on the respective host carrying a plasmid with the gene *up2* under the control of its native promoter or, as control, the *gfp* under the constitutive promoter P_{LAC}. Images are representative of two independent experiments. **b** Core phylogenetic tree for phages from the VIRIDIC family (>50% identities) including the helper phage Φ115 (red). The phylogenic tree is based on core proteins (25% identities and 80% coverage).

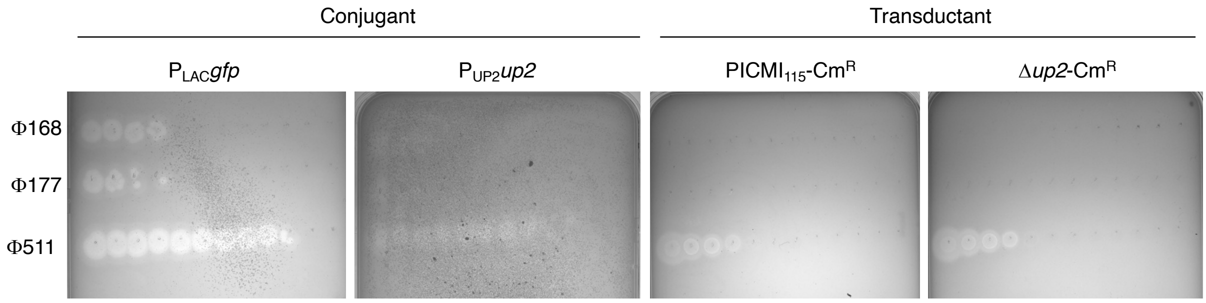

Supplementary Figure 19. Changes in susceptibility to phage killing observed for up2 expressing *V. chagasii* (conjugant) or carrying $PICMI_{115}$ (transductant). Tenfold dilutions of the indicated phages were spotted on V511 conjugants with plasmid containing the *gfp* under a constitutive promoter (P_{LAC}*gfp*) or the gene *up2* under the control of its native promoter and transductants carrying the full satellite ($PICMI_{115}$-Cm^R) or a derivative with an inactivated *up2* (Δ*up2*-Cm^R). Images are representative of two independent experiments.

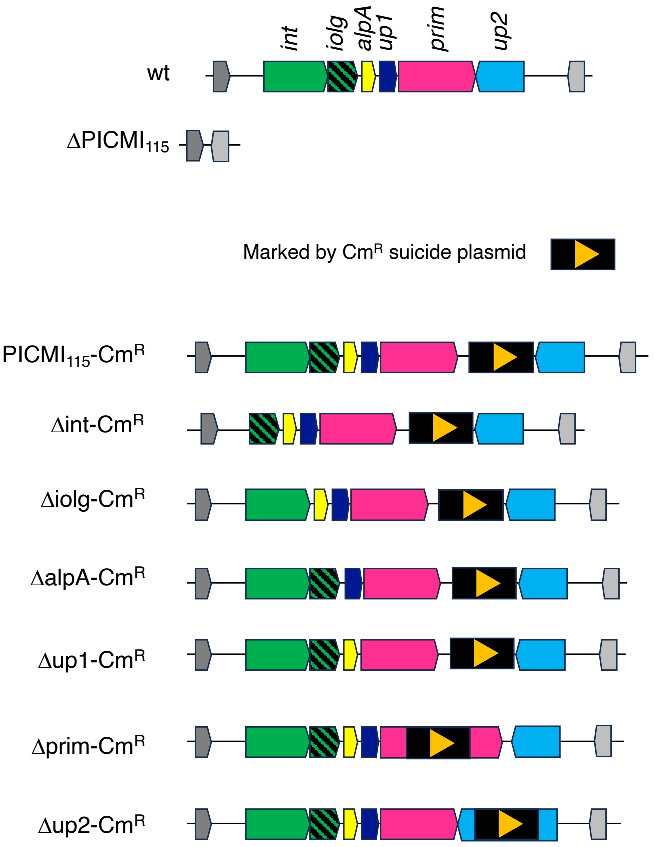

Supplementary Figure 20. Schematic representation of strategies for generating single-gene mutants
