# Supplementary material for: Phage-inducible chromosomal minimalist islands (PICMIs), a novel family of small marine satellites of virulent phages
Source: Nat Commun. 2024 Jan 22;15:664. doi: 10.1038/s41467-024-44965-1 (PMC10803314; doi:10.1038/s41467-024-44965-1)
Supplement: Supplementary file 3 — Description of Additional Supplementary Files [file 41467_2024_44965_MOESM3_ESM.pdf]

Description of Additional Supplementary Files:

Supplementary Data 1: Nanopore sequencing data

Supplementary Data 2: Search for PICMI using Genbank bacterial complete genomes database

Supplementary Data 3: Search for PICMI using NCBI Assembly database

Supplementary Data 4: Phages used in the present study

Supplementary Data 5: Vibrio chagasii strains (wild type) used in the present study

Supplementary Data 6: Strains used or established for the genefic approach

Supplementary Data 7: Primers used in this study

Supplementary Data 8: Plasmids used or established in the present study (stored in E. coli)

Supplementary Data 9: AlpA from the PICMI carried by V. chagasii and V. aestuarianus are interchangeable for PICMI excision

Supplementary Data 10: Alphafold analyses

Supplementary Software File: MacSyFinder models used to identify PICMI
