# Supplementary figures and images for: Phage-inducible chromosomal minimalist islands (PICMIs), a novel family of small marine satellites of virulent phages
Source: Nat Commun. 2024 Jan 22;15:664. doi: 10.1038/s41467-024-44965-1 (PMC10803314; doi:10.1038/s41467-024-44965-1)

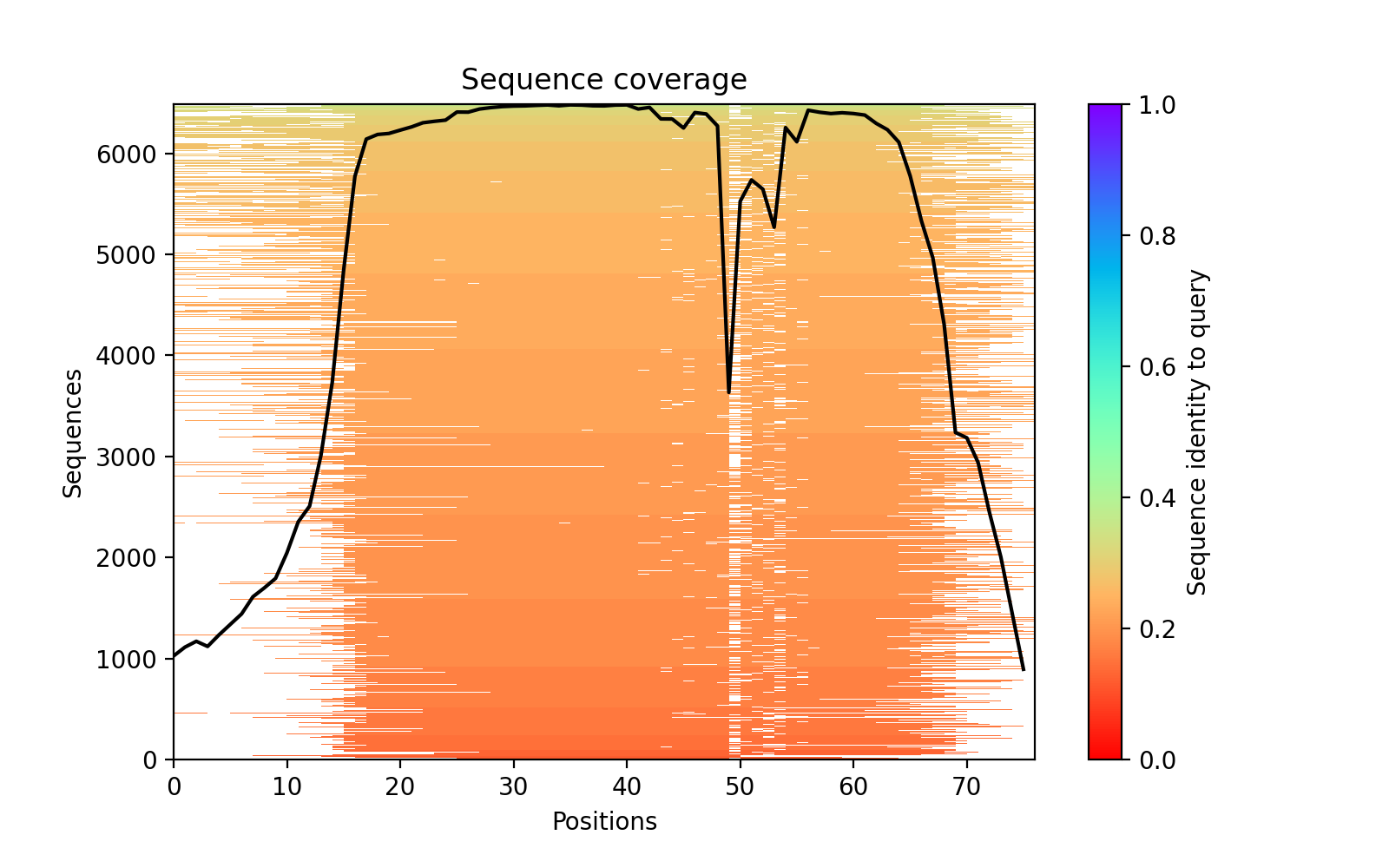

Supplement: Supplementary file 5 — Supplementary Data 10 [file 41467_2024_44965_MOESM5_ESM.zip › AlpA_34_P_115/AlpA_34_P_115_0b1e2_coverage.png]

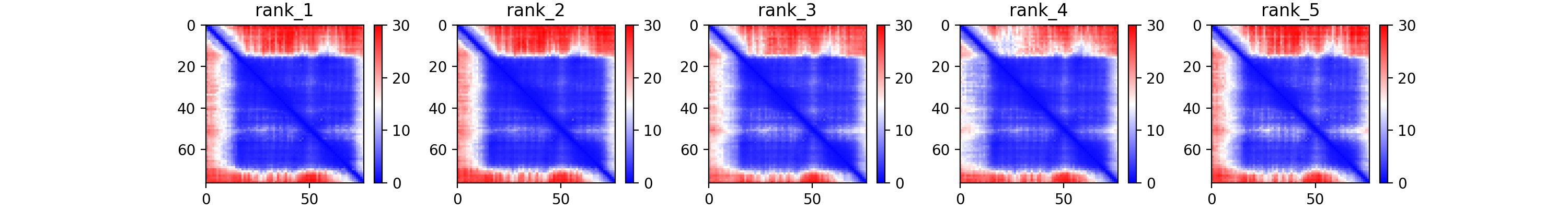

Supplement: Supplementary file 5 — Supplementary Data 10 [file 41467_2024_44965_MOESM5_ESM.zip › AlpA_34_P_115/AlpA_34_P_115_0b1e2_PAE.png]

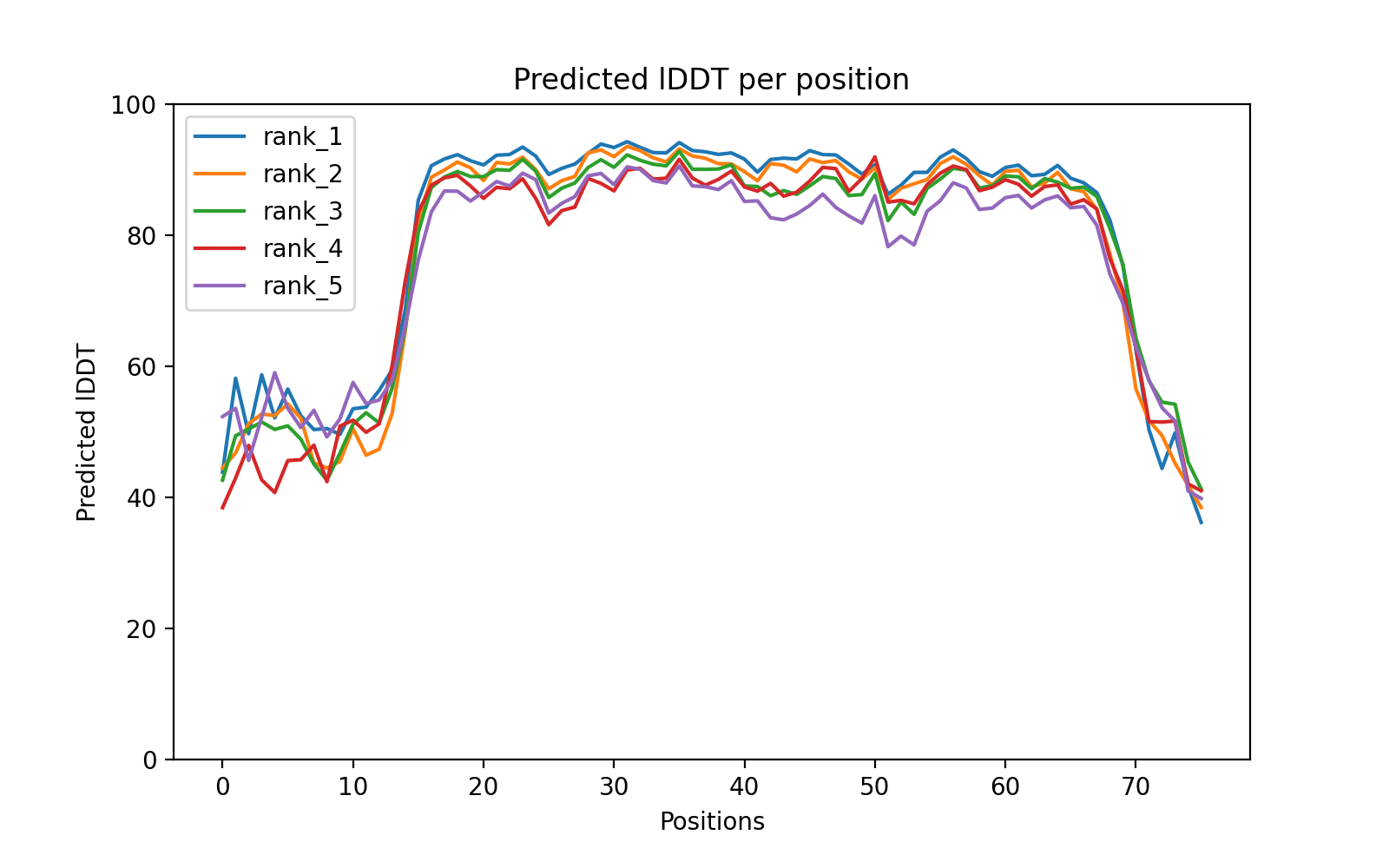

Supplement: Supplementary file 5 — Supplementary Data 10 [file 41467_2024_44965_MOESM5_ESM.zip › AlpA_34_P_115/AlpA_34_P_115_0b1e2_plddt.png]
